# Construction of a nomogram based on disinfection methods and clinical characteristics of ICU patients to forecast hospital-acquired respiratory infections: A single-center study from China

**DOI:** 10.1371/journal.pone.0331172

**Published:** 2025-08-29

**Authors:** Congjie Zhang, Yiyuan Zhang, Changyuan Quan, Xiaotao Lai, Sheng Ming, Hemin Zhang, Haiqun Wu, Fangfang He

**Affiliations:** 1 Administration Department of Nosocomial Infection, Hezhou People’s Hospital, Hezhou, Guangxi, China; 2 Department of pediatric, Hezhou People’s Hospital, Hezhou, Guangxi, China; Hokkaido University: Hokkaido Daigaku, JAPAN

## Abstract

**Background:**

Hospital-acquired respiratory tract infections (HARTI) are increasingly recognized by healthcare workers, especially among critically ill patients who are particularly susceptible. The selection of effective surface disinfectants can effectively block the transmission of pathogens, with chlorine-based disinfectants being widely used at present. This study constructs a nomogram by analyzing the choice of surface disinfection methods and clinical information of patients, to predict the occurrence of HARTI in ICU patients.

**Method:**

This study collected 592 patients admitted to the ICU from 01/01/2020–31/12/2023, and used binary logistic regression analysis to predict the predictive effect of Malignant tumor, Admission ICU unit, CRP, APTT, Any norepinephrine use, Blood.transfusion, Chlorine disinfectant, Tracheotomy on the occurrence of HARTI in ICU patients. And use R studio to construct nomogram model.

**Result:**

The results indicate that MONO (7.16[2.16,23.71]), BUN (0.24[0.06,0.88]), SOFA (4.5[1.48,13.74]), chlorine disinfectant (500 mg/L) (0.02[0,0.07]) in the choice of disinfection method, and bed railing (0.14[0.04,0.48]), micro-infusion pump (0.31[0.1,0.98]) in the area of disinfection are independent predictors of HARTI occurrence. The nomogram derived from the study demonstrated good predictive performance and showed minor errors in both the training and validation sets, providing significant clinical benefits to most patients. Subgroup analysis also well demonstrated this point, showing that it can better reduce the occurrence of HARTI patients in the ICU compared to two other types of disinfectants.

**Conclusion:**

Regulation of MONO and BUN values in blood indicators for ICU patients, intervention on corresponding indicators in the SOFA score, and the use of Chlorine disinfectant (500 mg/L) for surface disinfection, with a focus on disinfecting bed railings and micro-infusion pumps, can significantly reduce the incidence of HARTI, allow for early prevention and adjustment of HARI, and simultaneously benefit more patients.

## Introduction

Hospital-acquired infections (HAI) refer to infections acquired in hospitals, nursing homes, rehabilitation facilities, outpatient clinics, diagnostic laboratories, or other clinical settings. Many dynamic processes can introduce contaminants into these clinical environments [1,2]. Contaminated equipment, linens, airborne droplets, healthcare workers, and the skin microbiota of patients themselves can all become sources of HAI. ICU patients, due to the particularity of the population, often constitute a group prone to infections. Reports indicate that this year, the proportion of severely ill patients with immune deficiencies has risen to about one-third of all ICU admissions. The use of steroids and other immunosuppressants during hospital stays is likely to further increase this trend. A large number of ICU patients may survive for many years under conditions of immune deficiency, which will expose them to severe infection risks [3]. Hospital-acquired respiratory tract infections (HARTI), as a significant component of HAI, have a major impact on patient prognosis and subsequent treatment.

Intensive care unit (ICU) medical care includes three basic elements: firstly, the environment in which patient care is provided; secondly, the tools and equipment used; and lastly, the personnel circulating in these environments (including patients, staff, and family members) [4], each of which contributes to the risk of microbial contamination in the hospital environment [5]. Among existing disinfectants, chlorine-based and phenolic products are the most widely used in hospital environments [6,7], compared to alcohol-based disinfectant products, they have advantages such as suitability for large surfaces, longer persistence, and safety [8], and are often used for disinfecting surfaces in the ICU. However, to date, there has not been extensive research on the correlation between disinfection methods and hospital-acquired respiratory tract infections (HARTI) in ICU patients.

Therefore, this study utilized a single-center retrospective approach to collect data including patients’ blood test results at admission, medication and nursing care during hospitalization, and the choice of surface disinfectants used during the stay. Based on these clinical characteristics, a predictive model was constructed for ICU patients to forecast the occurrence of HARTI. The aim is to achieve early, simple, and convenient prediction, assisting clinical staff in making timely interventions and adjusting treatment plans accordingly.

## Materials and methods

### Patient section

This is a single-center retrospective cohort study. A total of 592 patients admitted to the ICU of Hezhou People’s Hospital from 01/01/2020 to 31/12/2023 were collected for this study. The information was collected from 01/04/2024 to 30/4/2024.

In this study, patients were male or female, aged ≥18 and ≤90 years old, admitted to our hospital’s ICU, with relevant clinical information available. The criteria for HARTI (Hospital-Acquired Respiratory Tract Infections) were determined as follows: (1) Signs and symptoms appearing after ≥48 hours of admission to an acute or chronic medical facility or within <7 days after discharge, including criteria 3, 5, and 6; (2) Chest X-ray or CT scan obtained within 48 hours after ICU admission showing no new infiltrates or progressively worsening infiltrates; (3) Respiratory samples for Gram stain and culture obtained within 48 hours before screening and after the onset of HARTI symptoms (before systemic antibiotic treatment (For infections where the pathogen is not yet identified, empirical broad-spectrum antibiotic treatment is often used after sampling and culturing the patient’s bodily fluids. Once the bacterial culture results are clear, antibiotics are administered based on the results of the bacterial drug susceptibility tests)); (4) At least one of the following systemic signs: A. Fever (body temperature >38°C) or hypothermia (rectal/core temperature <35°C); B. White blood cell (WBC) count >10000/mm^3^, or WBC count <4500/mm^3^, or band forms >15%; (5) At least two of the following respiratory signs or symptoms: A. New onset of cough (or worsening of an existing cough); B. Production of purulent sputum or tracheal secretions; C. Auscultatory findings consistent with pneumonia/consolidation (e.g., crackles, dry rales, bronchial breath sounds, dullness on percussion, bronchophony); D. Difficulty breathing, shortness of breath, or insufficient oxygenation (O2 saturation <90% or PaO2 < 60mmHg when breathing room air); E. Need for mechanical ventilation, or for subjects already on mechanical ventilation, emergency modification of the ventilatory support system to enhance oxygenation due to worsening arterial blood gases or PaO2/FiO2. Exclusion criteria were as follows: (1) Patients with other medical or psychiatric conditions; (2) Patients with concurrent pulmonary diseases that could interfere with the assessment of treatment response (including but not limited to lung cancer, active tuberculosis, cystic fibrosis, granulomatous diseases, fungal lung infections, or recent pulmonary embolism prior to ICU admission); (3) Patients with lung abscess, empyema, or obstructive pneumonia prior to ICU admission; (4) Patients with severe immunodeficiency diseases (such as leukemia, AIDS, and other related diseases); (5) Before the occurrence of HARTI, the patient had already been using antibiotics to combat infection. This study included ICU patients from Hezhou People’s Hospital who met the above criteria and divided them into a training set and a validation set at a ratio of 7:3 for the construction and validation of a nomogram.

This study has been approved by the Ethics Committee of Hezhou People’s Hospital (Ethics Approval Number: 2023061068) in accordance with the Declaration of Helsinki. All healthcare workers involved in the management of ICU patients have undergone comprehensive and systematic training in hospital infection prevention, with specific programs fully adhering to the national health industry standards (WS/T 509–5016, WS/T 512–2016). The data extracted were provided by the hospital’s internal database after de-identification of sensitive information, and the same Ethics Committee waived the requirement for informed consent.

### Data collection and definition

This study collected data on patients’ clinical characteristics, baseline clinical data, methods and scope of disinfection, as well as results from routine blood tests, blood biochemistry, and coagulation function tests. Baseline clinical data included gender, age, height, weight, history of hepatitis, history of alcohol consumption, and smoking history.

Additionally, this study collected patients’ test results upon admission, which included the following data: White blood cell count (WBC), Red blood cell count (RBC), Hemoglobin (Hb), Hematocrit (HCT), Mean corpuscular volume (MCV), Mean corpuscular hemoglobin concentration (MCHC), Neutrophils (NEU), Lymphocytes (LYM), Monocytes (MONO), Eosinophils (EOS), Basophils (BAS), Albumin (ALB), Platelets (PLT), Platelet hematocrit (PCT), C-Reactive Protein (CRP), Aspartate aminotransferase (AST), Alanine aminotransferase (ALT), Albumin (ALB), High-density lipoprotein (HDL), Low-density lipoprotein (LDL), Triglycerides (TG), Gamma-glutamyltransferase (GGT), Total bilirubin (TBIL), Creatinine (Cr), Blood urea nitrogen (BUN), Prothrombin time (PT), Activated partial thromboplastin time (APTT), Fibrinogen (FIB), D-Dimer, and other relevant data. The clinical characteristics of the patients collected included the following data: hypertension, diabetes, heart disease, renal insufficiency, brain diseases, malignant tumors, initial SOFA score upon admission, initial APACHE II score upon admission, choice of surface disinfectant (We use “1” to represent that the corresponding measures have been taken, and “0” to indicate that the corresponding measures have not been taken), area of surface disinfection (The frequency of disinfectant use can refer to the Chinese health industry practitioner standards (WS/T 512–2016))(We use “1” to represent that the area have been disinfected, and “0” to indicate that the area have not been disinfected), treatment measures, and other related conditions. Additionally, we have supplemented the collection of bacterial culture results from respiratory sputum specimens of HARTI patients and conducted a subgroup analysis, which includes 8 types of bacteria: Haemophilus influenzae (Hin), Streptococcus pneumoniae (Spn), Klebsiella pneumoniae (Kpn), Pseudomonas aeruginosa (Pae), Stenotrophomonas maltophilia (Pma), Acinetobacter baumannii (Aba), Staphylococcus aureus (Sau), Escherichia coli (Eco).

To assess the correlation between the disinfection status of object surfaces and patient morbidity, we observed the environment most frequently in contact with patients during their ICU stay and utilized a non-probability purposive sample. The researchers determined the study parameters based on the research objectives [9–11]. The selection criteria were object surfaces with a high frequency of contact during the patient’s ICU stay, as they pose a higher risk of contamination. The study ultimately focused on the following three object surfaces: bed railing, Electrocardiogram monitor, Micro-infusion pump.

### Outcome

This study considers the occurrence of HARTI during hospitalization as the outcome event, with diagnostic criteria referring to the standards mentioned in section 2.1. A positive occurrence of HARTI is indicated by its presence, while its absence is indicated as negative.

### Statistical analysis

In this study, data were first aggregated and then split into training and validation sets in a 7:3 ratio using SPSS 21.0 (SPSS Inc., Chicago, IL). Continuous variables collected were dichotomized into two groups based on their means: values less than the mean were defined as group 0, and values greater than the mean were defined as group 1. Clinical data and test results from the training set were compared between the HARTI group and the No HARTI group using the Kruskal-Wallis test. Categorical data were described using frequencies (percentages) and compared using the Chi-squared test or Fisher’s exact test.

GraphPad Prism 9.5.0 was utilized to plot the ROC curves for variables including MONO, BUN, SOFA, Chlorine disinfectant (500 mg/L), Bed railing, Micro-infusion pump, calculating the area under the curve (AUC). A p-value of less than 0.05 in a two-tailed test was considered statistically significant.

Subsequently, univariate logistic regression analyses were performed using SPSS to identify clinically significant factors, which were then included in a multivariate logistic regression analysis to identify independent predictors of HARTI.

Finally, based on the results of the multivariate logistic regression analysis, a nomogram of the independent predictors was created using R Studio (version 4.2.2). The predictive performance of the model was evaluated using the ROC curve and AUC. The model’s accuracy was checked with a calibration plot to find the average error, and the model’s usefulness in a clinical setting was examined using a Decision Curve Analysis (DCA).

## Result

### Clinical factors and their predictive relationship with HARTI

**Fig 1:**
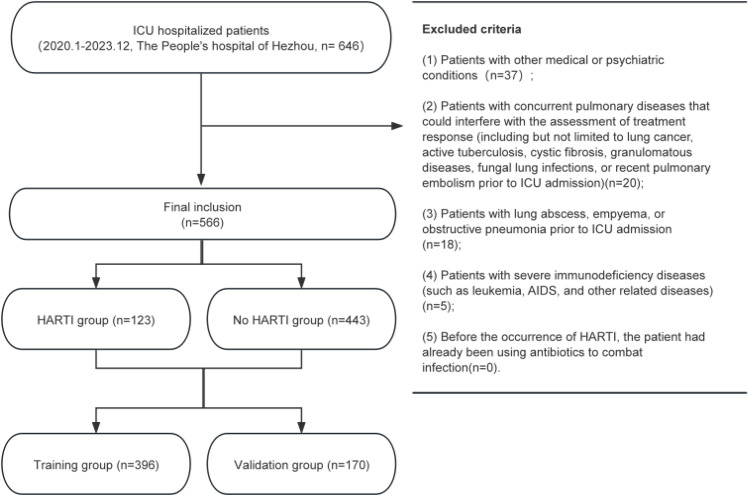
The flow chart of this study.

This study collected data from 646 patients, with 80 patients excluded due to various factors, ultimately including 566 patients in a retrospective study, as shown in Fig 1. All data were compiled and categorized based on whether HARTI occurred after ICU admission, defining those diagnosed with HARTI as the HARTI group, totaling 123 patients, and those not diagnosed with HARTI as the No HARTI group, totaling 443 patients. The collected data were randomly sampled at a ratio of 7:3, dividing into a training group of 396 patients and a validation group of 170 patients. Baseline data between the two groups were compared, as shown in Table 1. In the Training group, there were 117 females (29.55%) and 279 males (70.45%), with histories of smoking, alcohol abuse, hepatitis, hypertension, diabetes, heart disease, brain disease, and malignant tumors in 83 (20.96%), 58 (14.65%), 24 (6.06%), 155 (39.14%), 57 (14.39%), 185 (46.72%), 160 (40.4%), and 45 (11.36%) patients, respectively. There was no statistical difference in baseline data between groups (p > 0.05), indicating comparability.

**Table 1:** Baseline data table for comparison of training and validation group.

Testvar	Training (n = 396)	Validation (n = 170)	p
Sex			0.753
Female	117 (29.55%)	48 (28.24%)	
Male	279 (70.45%)	122 (71.76%)	
Age	61 (51,71)	62 (53,73)	0.213
Height	161 (155,166)	161 (155.25,166)	0.876
Weight	63 (55,70)	61 (55,69)	0.269
BMI	24.27 (21.29,27.2)	23.84 (20.94,26.3)	0.260
Smoke			0.256
No	313 (79.04%)	127 (74.71%)	
Yes	83 (20.96%)	43 (25.29%)	
Alcoholism			0.213
No	338 (85.35%)	138 (81.18%)	
Yes	58 (14.65%)	32 (18.82%)	
Hepatitis			0.522
No	372 (93.94%)	162 (95.29%)	
Yes	24 (6.06%)	8 (4.71%)	
Hypertension			0.162
No	241 (60.86%)	114 (67.06%)	
Yes	155 (39.14%)	56 (32.94%)	
Diabetes			0.648
No	339 (85.61%)	148 (87.06%)	
Yes	57 (14.39%)	22 (12.94%)	
Heart disease			0.278
No	211 (53.28%)	99 (58.24%)	
Yes	185 (46.72%)	71 (41.76%)	
Cerebrovasular disease			0.052
No	236 (59.6%)	116 (68.24%)	
Yes	160 (40.4%)	54 (31.76%)	
Malignant tumor			0.196
No	351 (88.64%)	144 (84.71%)	
Yes	45 (11.36%)	26 (15.29%)	

All sample data were dichotomized based on means, and the training group samples were compared between groups, categorized by the occurrence of HARTI. Statistical differences were found in Malignant tumor, HCT, MCHC, MONO, CRP, APTT, D.Dimer, ALB, Cr, BUN, APACHE II, SOFA, Chlorine disinfectant (500 mg/L), Alcohol(75%), Quaternary ammonium salt disinfectant wipes (2500 mg/L), Bed railing, Micro-infusion pump, Ventilation, Tracheotomy, Gastric tube (p < 0.05), as shown in Table 2.

**Table 2:** Data table for comparison of No HARTI and HARTI group.

Variable	No HARTI group (n = 310)	HARTI group (n = 86)	p
**Baseline data**			
Sex			0.087
Female	98 (31.61%)	19 (22.09%)	
Male	212 (68.39%)	67 (77.91%)	
Age			0.084
>60.39	144 (46.45%)	49 (56.98%)	
≤60.39	166 (53.55%)	37 (43.02%)	
Height			0.742
>160.67	154 (49.68%)	41 (47.67%)	
≤160.67	156 (50.32%)	45 (52.33%)	
Weight			0.559
>62.30	148 (47.74%)	38 (44.19%)	
≤62.30	162 (52.26%)	48 (55.81%)	
BMI			0.109
>24.22	160 (51.61%)	36 (41.86%)	
≤24.22	150 (48.39%)	50 (58.14%)	
Smoke			0.136
No	250 (80.65%)	63 (73.26%)	
Yes	60 (19.35%)	23 (26.74%)	
Alcoholism			0.062
No	270 (87.1%)	68 (79.07%)	
Yes	40 (12.9%)	18 (20.93%)	
Hepatitis			0.914
No	291 (93.87%)	81 (94.19%)	
Yes	19 (6.13%)	5 (5.81%)	
Hypertension			0.869
No	188 (60.65%)	53 (61.63%)	
Yes	122 (39.35%)	33 (38.37%)	
Diabetes			0.829
No	266 (85.81%)	73 (84.88%)	
Yes	44 (14.19%)	13 (15.12%)	
Heart disease			0.206
No	160 (51.61%)	51 (59.3%)	
Yes	150 (48.39%)	35 (40.7%)	
Cerebrovasula disease			0.495
No	182 (58.71%)	54 (62.79%)	
Yes	128 (41.29%)	32 (37.21%)	
Malignant tumor			0.027
No	269 (86.77%)	82 (95.35%)	
Yes	41 (13.23%)	4 (4.65%)	
**Laboratory parameter**			
RBC			0.135
>3.83	158 (50.97%)	36 (41.86%)	
≤3.83	152 (49.03%)	50 (58.14%)	
Hb			0.054
>108.41	159 (51.29%)	34 (39.53%)	
≤108.41	151 (48.71%)	52 (60.47%)	
HCT			0.039
>33.26	158 (50.97%)	33 (38.37%)	
≤33.26	152 (49.03%)	53 (61.63%)	
MCV			0.804
>87.74	118 (38.06%)	34 (39.53%)	
≤87.74	192 (61.94%)	52 (60.47%)	
MCHC			0.028
>325.46	142 (45.81%)	28 (32.56%)	
≤325.46	168 (54.19%)	58 (67.44%)	
WBC			0.342
>13.49	180 (58.06%)	45 (52.33%)	
≤13.49	130 (41.94%)	41 (47.67%)	
NEU			0.373
>11.9	186 (60%)	47 (54.65%)	
≤11.9	124 (40%)	39 (45.35%)	
LYM			0.201
>1.08	193 (62.26%)	47 (54.65%)	
≤1.08	117 (37.74%)	39 (45.35%)	
MONO			0.007
>0.70	204 (65.81%)	43 (50%)	
≤0.70	106 (34.19%)	43 (50%)	
EOS			0.357
>0.06	235 (75.81%)	61 (70.93%)	
≤0.06	75 (24.19%)	25 (29.07%)	
BAS			0.22
>0.02	178 (57.42%)	43 (50%)	
≤0.02	132 (42.58%)	43 (50%)	
PLT			0.94
>209.10	168 (54.19%)	47 (54.65%)	
≤209.10	142 (45.81%)	39 (45.35%)	
PCT			0.767
>0.19	153 (49.35%)	44 (51.16%)	
≤0.19	157 (50.65%)	42 (48.84%)	
CRP			0.042
>48.68	210 (67.74%)	68 (79.07%)	
≤48.68	100 (32.26%)	18 (20.93%)	
PT			0.119
>17.19	256 (82.58%)	77 (89.53%)	
≤17.19	54 (17.42%)	9 (10.47%)	
APTT			0.016
>37.05	215 (69.35%)	71 (82.56%)	
≤37.05	95 (30.65%)	15 (17.44%)	
FIB			0.146
>3.29	186 (60%)	59 (68.6%)	
≤3.29	124 (40%)	27 (31.4%)	
D.Dimer			0.006
>5.21	255 (82.26%)	59 (68.6%)	
≤5.21	55 (17.74%)	27 (31.4%)	
ALB			0.036
>33.41	155 (50%)	32 (37.21%)	
≤33.41	155 (50%)	54 (62.79%)	
HDL			0.317
>1.09	195 (62.9%)	49 (56.98%)	
≤1.09	115 (37.1%)	37 (43.02%)	
LDL			0.466
>2.17	207 (66.77%)	61 (70.93%)	
≤2.17	103 (33.23%)	25 (29.07%)	
TG			0.354
>1.59	230 (74.19%)	68 (79.07%)	
≤1.59	80 (25.81%)	18 (20.93%)	
GGT			0.168
>60.09	230 (74.19%)	70 (81.4%)	
≤60.09	80 (25.81%)	16 (18.6%)	
ALT			0.913
>59.09	258 (83.23%)	72 (83.72%)	
≤59.09	52 (16.77%)	14 (16.28%)	
AST			0.371
>124.90	262 (84.52%)	76 (88.37%)	
≤124.90	48 (15.48%)	10 (11.63%)	
TBIL			0.371
>20.25	254 (81.94%)	74 (86.05%)	
≤20.25	56 (18.06%)	12 (13.95%)	
Cr			0.031
>163.67	246 (79.35%)	77 (89.53%)	
≤163.67	64 (20.65%)	9 (10.47%)	
BUN			0.033
>10.20	204 (65.81%)	67 (77.91%)	
≤10.20	106 (34.19%)	19 (22.09%)	
**Severity of disease**			
APACHE II			<0.001
>28.54	155 (50%)	22 (25.58%)	
≤28.54	155 (50%)	64 (74.42%)	
SOFA			<0.001
>6.13	187 (60.32%)	28 (32.56%)	
≤6.13	123 (39.68%)	58 (67.44%)	
**Type of disinfectant**			
Chlorine disinfectant (500 mg/L)			<0.001
No	66 (21.29%)	72 (83.72%)	
Yes	244 (78.71%)	14 (16.28%)	
Alcohol(75%)			<0.001
No	291 (93.87%)	56 (65.12%)	
Yes	19 (6.13%)	30 (34.88%)	
Quaternary ammonium salt disinfectant wipes (2500 mg/L)			<0.001
No	244 (84.14%)	44 (51.16%)	
Yes	46 (15.86%)	42 (48.84%)	
**Disinfection range**			
Bed railing			<0.001
No	63 (20.32%)	36 (41.86%)	
Yes	247 (79.68%)	50 (58.14%)	
Electrocardiogram monitor			0.075
No	143 (46.13%)	49 (56.98%)	
Yes	167 (53.87%)	37 (43.02%)	
Micro-infusion pump			0.004
No	148 (47.74%)	56 (65.12%)	
Yes	162 (52.26%)	30 (34.88%)	
**Treatment interventions before HARTI events**			
Ventilation			<0.001
No	94 (30.32%)	3 (3.49%)	
Yes	216 (69.68%)	83 (96.51%)	
Tracheotomy			<0.001
No	273 (88.06%)	39 (45.35%)	
Yes	37 (11.94%)	47 (54.65%)	
Catheter			0.112
No	13 (4.19%)	0 (0%)	
Yes	297 (95.81%)	86 (100%)	
Dialysis			0.121
No	265 (85.48%)	79 (91.86%)	
Yes	45 (14.52%)	7 (8.14%)	
Gastric tube			<0.001
No	159 (51.29%)	2 (2.33%)	
Yes	151 (48.71%)	84 (97.67%)	

**Fig 2:**
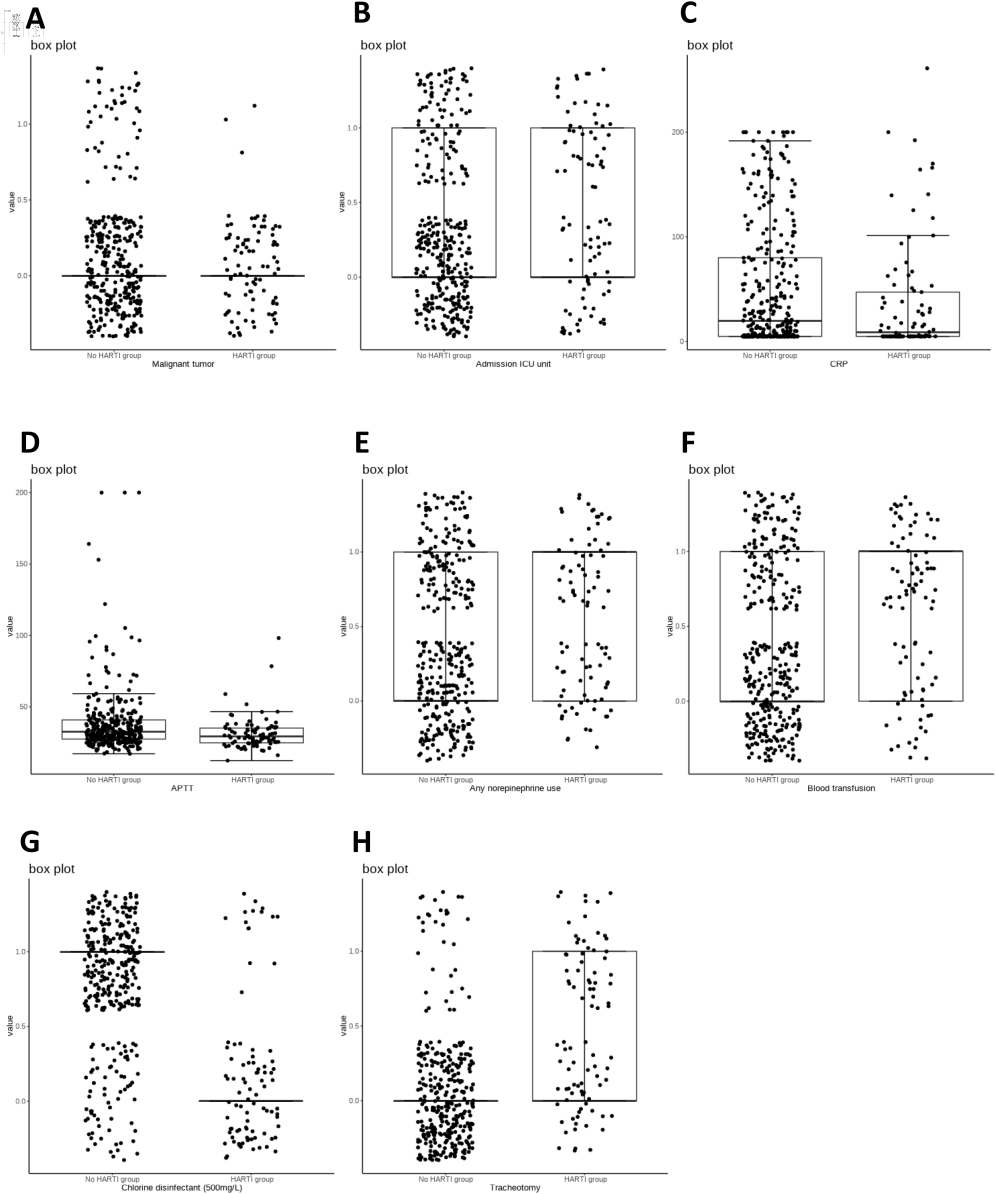
Box plot of risk factors. A the box plot of Malignant tumor; B the box plot of Adimission ICU unit; C the box plot of CRP; D the box plot of APTT; E the box plot of Any neropinephrine use; F the box plot of Blood transfusion; G the box plot of Chlorine disinfectant; H the box plot of Tracheotomy.

**Fig 3:**
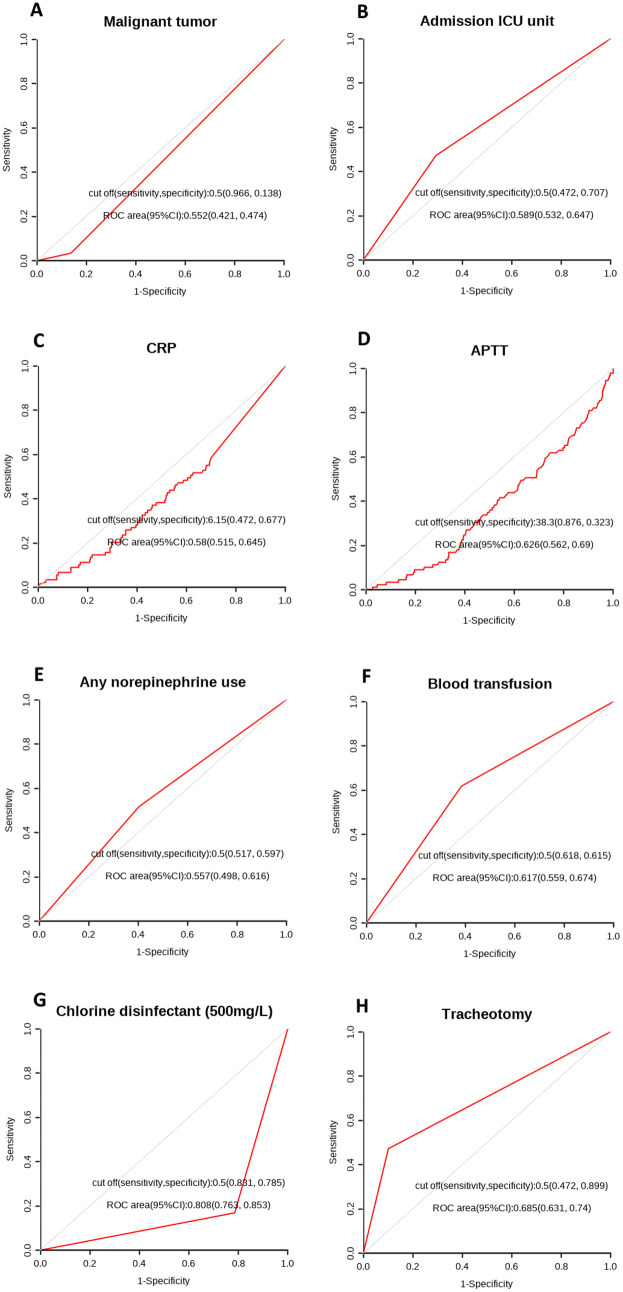
ROC curve of risk factors. A the ROC curve of Malignant tumor; B the ROC curve of Adimission ICU unit; C the ROC curve of CRP; D the ROC curve of APTT; E the ROC curve of Any neropinephrine use; F the ROC curve of Blood transfusion; G the ROC curve of Chlorine disinfectant; H the ROC curve of Tracheotomy.

Subsequently, box plots for predicting HARTI for MONO, BUN, SOFA, Chlorine disinfectant (500 mg/L), Bed railing, Micro-infusion pump (Fig 2A–F) and ROC curves (Fig 3A–F) were drawn, calculating the optimal cutoff values, sensitivity, and specificity. The results showed that the optimal cutoff value for SOFA was 6.5, with a sensitivity of 0.647 and specificity of 0.603; for Chlorine disinfectant (500 mg/L), the optimal cutoff value was 0.5, with a sensitivity of 0.837 and specificity of 0.787; for Bed Railing, the optimal cutoff value was 0.5, with a sensitivity of 0.419 and specificity of 0.797; for Micro-infusion pump, the optimal cutoff value was 0.5, with a sensitivity of 0.651 and specificity of 0.523; for MONO, the optimal cutoff value was 0.655, with a sensitivity of 0.581 and specificity of 0.59; for BUN, the optimal cutoff value was 12.33, with a sensitivity of 0.919 and specificity of 0.287. Box plots were also drawn to visually display the distribution differences between the two groups (Fig 2A–F).

Clinical data were subjected to univariate binary logistic regression analysis, with results shown in Table 3. Factors such as malignant tumor (0.32 [0.11, 0.92]), HCT (1.67 [1.02, 2.72]), MCHC (1.75 [1.06, 2.9]), MONO (1.92 [1.19, 3.12]), CRP (0.56 [0.31, 0.98]), APTT (0.48 [0.26, 0.88]), D.Dimer (2.12 [1.24, 3.64]), ALB (1.69 [1.03, 2.76]), Cr (0.45 [0.21, 0.94]), BUN (0.55 [0.31, 0.96]), APACHE II (2.91 [1.71, 4.96]), SOFA (3.15 [1.9, 5.22]), chlorine disinfectant (500 mg/L) (0.05 [0.03, 0.1]), alcohol (75%) (8.2 [4.32, 15.59]), quaternary ammonium salt disinfectant wipes (2500 mg/L) (5.06 [2.99, 8.58]), bed railing (0.35 [0.21, 0.59]), micro-infusion pump (0.49 [0.3, 0.8]), ventilation (12.04 [3.71, 39.07]), tracheotomy (8.89 [5.15, 15.35]), and gastric tube (44.23 [10.69, 182.94]) were identified as risk factors for HARTI within the ICU. Factors with significance in the univariate analysis were then included in a multivariate binary logistic regression analysis using a stepwise method. The results identified MONO (7.16 [2.16, 23.71]), BUN (0.24 [0.06, 0.88]), SOFA (4.5 [1.48, 13.74]), chlorine disinfectant (500 mg/L) (0.02 [0, 0.07]), bed railing (0.14 [0.04, 0.48]), and micro-infusion pump (0.31 [0.1, 0.98]) as independent predictors, as shown in Table 4.

**Table 3:** Single binary logistic regression analysis results.

Variable	OR[95%CI]	p
**Baseline data**		
Sex		0.089
Female	1.63[0.93,2.86]	
Male	1	
Age		0.085
>60.39	0.66[0.4,1.06]	
≤60.39	1	
Height		0.742
>160.67	1.08[0.67,1.75]	
≤160.67	1	
Weight		0.559
>62.30	1.15[0.71,1.87]	
≤62.30	1	
BMI		0.111
>24.22	1.48[0.91,2.4]	
≤24.22	1	
Smoke		0.138
No	1.52[0.87,2.65]	
Yes	1	
Alcoholism		0.065
No	1.79[0.96,3.31]	
Yes	1	
Hepatitis		0.914
No	0.95[0.34,2.61]	
Yes	1	
Hypertension		0.869
No	0.96[0.59,1.57]	
Yes	1	
Diabetes		0.829
No	1.08[0.55,2.11]	
Yes	1	
Heart disease		0.207
No	0.73[0.45,1.19]	
Yes	1	
Cerebrovasular disease		0.495
No	0.84[0.52,1.38]	
Yes	1	
Malignant tumor		0.034
No	0.32[0.11,0.92]	
Yes	1	
**Laboratory parameter**		
RBC		0.136
>3.83	1.44[0.89,2.34]	
≤3.83	1	
Hb		0.055
>108.41	1.61[0.99,2.62]	
≤108.41	1	
HCT		0.04
>33.26	1.67[1.02,2.72]	
≤33.26	1	
MCV		0.804
>87.74	0.94[0.58,1.53]	
≤87.74	1	
MCHC		0.029
>325.46	1.75[1.06,2.9]	
≤325.46	1	
WBC		0.342
>13.49	1.26[0.78,2.04]	
≤13.49	1	
NEU		0.373
>11.9	1.24[0.77,2.01]	
≤11.9	1	
LYM		0.202
>1.08	1.37[0.84,2.22]	
≤1.08	1	
MONO		0.008
>0.70	1.92[1.19,3.12]	
≤0.70	1	
EOS		0.358
>0.06	1.28[0.75,2.19]	
≤0.06	1	
BAS		0.221
>0.02	1.35[0.84,2.18]	
≤0.02	1	
PLT		0.94
>209.10	0.98[0.61,1.59]	
≤209.10	1	
PCT		0.767
>0.19	0.93[0.58,1.5]	
≤0.19	1	
CRP		0.044
>48.68	0.56[0.31,0.98]	
≤48.68	1	
PT		0.123
>17.19	0.55[0.26,1.17]	
≤17.19	1	
APTT		0.017
>37.05	0.48[0.26,0.88]	
≤37.05	1	
FIB		0.147
>3.29	0.69[0.41,1.14]	
≤3.29		
D.Dimer		0.006
>5.21	2.12[1.24,3.64]	
≤5.21	1	
ALB		0.037
>33.41	1.69[1.03,2.76]	
≤33.41	1	
HDL		0.318
>1.09	1.28[0.79,2.08]	
≤1.09	1	
LDL		0.466
>2.17	0.82[0.49,1.39]	
≤2.17	1	
TG		0.355
>1.59	0.76[0.43,1.36]	
≤1.59	1	
GGT		0.17
>60.09	0.66[0.36,1.2]	
≤60.09	1	
ALT		0.913
>59.09	0.96[0.51,1.84]	
≤59.09	1	
AST		0.373
>124.90	0.72[0.35,1.49]	
≤124.90	1	
TBIL		0.372
>20.25	0.74[0.37,1.44]	
≤20.25	1	
Cr		0.035
>163.67	0.45[0.21,0.94]	
≤163.67	1	
BUN		0.034
>10.20	0.55[0.31,0.96]	
≤10.20	1	
**Severity of disease**		
APACHE II		<0.001
>28.54	2.91[1.71,4.96]	
≤28.54	1	
SOFA		<0.001
>6.13	3.15[1.9,5.22]	
≤6.13	1	
**Type of disinfectant**		
Chlorine disinfectant (500 mg/L)		<0.001
No	0.05[0.03,0.1]	
Yes	1	
Alcohol(75%)		<0.001
No	8.2[4.32,15.59]	
Yes	1	
Quaternary ammonium salt disinfectant wipes (2500 mg/L)		<0.001
No	5.06[2.99,8.58]	
Yes	1	
**Disinfection range**		
Bed railing		<0.001
No	0.35[0.21,0.59]	
Yes	1	
Electrocardiogram monitor		0.076
No	0.65[0.4,1.05]	
Yes	1	
Micro-infusion pump		0.005
No	0.49[0.3,0.8]	
Yes	1	
**Treatment interventions before HARTI events**		
Ventilation		<0.001
No	12.04[3.71,39.07]	
Yes	1	
Tracheotomy		<0.001
No	8.89[5.15,15.35]	
Yes	1	
Catheter		0.982
No	9.81[4.51,16.01]	
Yes	1	
Dialysis		0.127
No	0.52[0.23,1.2]	
Yes	1	
Gastric tube		<0.001
No	44.23[10.69,182.94]	
Yes	1	

**Table 4:** Multivariate binary logistic regression analysis results.

Variable	OR[95%CI]	p
MONO		0.001
>0.70	7.16[2.16,23.71]	
≤0.70	1	
CRP		0.05
>48.68	0.3[0.09,1]	
≤48.68	1	
APTT		0.161
>37.05	0.39[0.11,1.45]	
≤37.05	1	
BUN		0.032
>10.20	0.24[0.06,0.88]	
≤10.20	1	
APACHE_II		0.073
>28.54	2.82[0.91,8.76]	
≤28.54	1	
SOFA		0.008
>6.13	4.5[1.48,13.74]	
≤6.13	1	
Chlorine disinfectant (500 mg/L)		<0.001
No	0.02[0,0.07]	
Yes	1	
Alcohol(75%)		0.991
No	0.15[0.01,0.52]	
Yes	1	
Bed railing		0.002
No	0.14[0.04,0.48]	
Yes	1	
Micro-infusion pump		0.047
No	0.31[0.1,0.98]	
Yes	1	
Ventilation		0.103
No	10.46[0.62,176.11]	
Yes	1	
Tracheotomy		0.076
No	2.79[0.9,8.69]	
Yes	1	
Gastric tube		0.989
No	11.25[0.53,181.22]	
Yes	1	

### Construction and evaluation of nomograms

**Fig 4:**
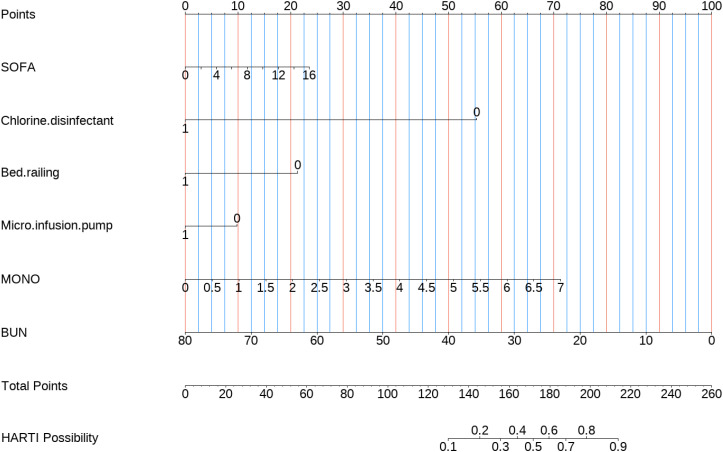
Nomogram of clinical data.

**Fig 5:**
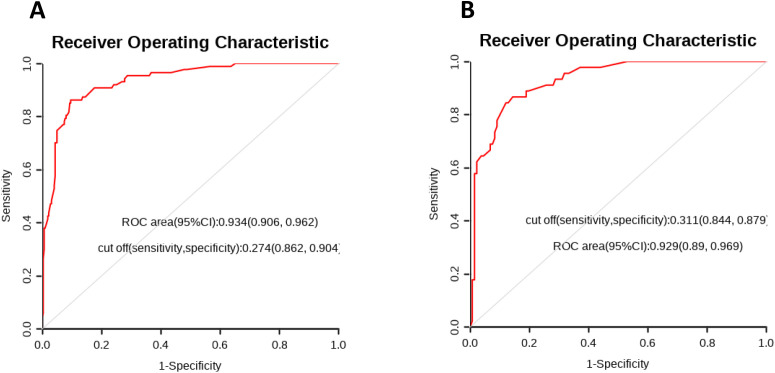
ROC curve of data. A ROC curve of training group; B ROC curve of validation group.

**Fig 6:**
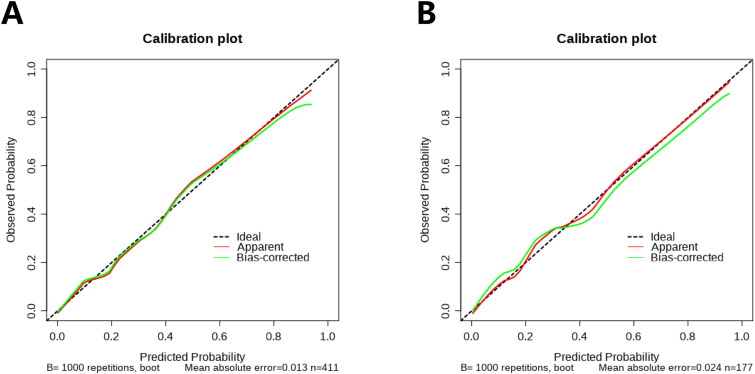
Calibration curve of data. A Calibration curve of training set data; B Calibration curve of validation set data.

**Fig 7:**
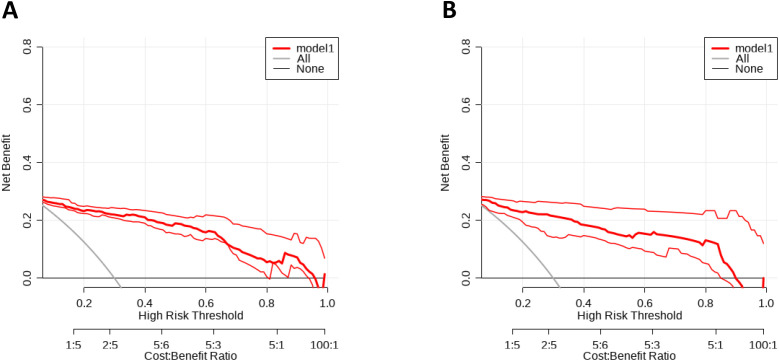
DCA curve of clinical data. A DCA curve of training data set; B DCA curve of validation data set.

To further validate the predictive capability of various factors on HARTI patients within the ICU, a nomogram model as shown in Fig 4 was constructed using R studio software based on the results of the multivariate analysis (MONO, BUN, SOFA, Chlorine disinfectant (500 mg/L), Bed railing, Micro-infusion pump). The nomogram was developed with the training group data, as shown in Fig 5. The predictive performance of this nomogram was verified by plotting the ROC curve for the training group (Fig 5A), which yielded a C-index of 0.885, indicating good predictive performance. The calibration curve for the training group (Fig 6A) suggested an average error of 0.028. The decision curve analysis (DCA) for the training group (Fig 7A) indicated good clinical benefit within a threshold range of 0.01–0.97. The model was also validated using the validation group data, with the ROC curve for the validation group (Fig 5B) showing a C-index of 0.897. The calibration curve for the validation group (Fig 6B) indicated an average error of 0.028, and the DCA (Fig 7B) showed good clinical benefit within a threshold range of 0.02–0.92.

Taking patient 6 from the training set as an example, the patient’s SOFA score is 8, MONO is 1, and BUN is 5.01. The method used for disinfecting object surfaces is quaternary ammonium wipes, and the bed rails and micro-infusion pump have been included in the disinfection range. The final patient score is 205.5, corresponding to a HARTI occurrence probability of >80%, which aligns with the patient’s corresponding final outcome event.

It is evident that the nomogram demonstrated good predictive performance, minimal error, and potential clinical benefit for a majority of patients, both in the training and validation groups.

### Subgroup analysis of HARTI patients under different disinfection methods

We collected the bacterial culture results of HARTI patients under different disinfection methods, and the results are presented in tabular form. Among all the collected samples, there were 123 HARTI patients, of which 19 patients still developed HARTI after disinfection with Chlorine disinfectant (500 mg/L) (Table 5). Among these, 8 cases (42.11%) were infected with Hin, 5 cases (26.32%) with Spn, 2 cases (10.53%) with Aba, 2 cases (10.53%) with Kpn, and 2 cases (10.53%) with Pae. A total of 45 patients still developed HARTI after disinfection with Alcohol (75%) (Table 6), including 15 cases of Hin (33.33%), 2 cases of Sau (4.44%), 11 cases of Spn (24.44%), 1 case of Eco (2.22%), 1 case of Aba (2.22%), 10 cases of Kpn (22.22%), 4 cases of Pae (8.89%), and 1 case of Pma (2.22%). A total of 59 patients still developed HARTI after disinfection with Quaternary ammonium salt disinfectant wipes (2500 mg/L) (Table 7), including 18 cases of Hin (30.51%), 1 case of Sau (1.69%), 23 cases of Spn (39.98%), 1 case of Eco (1.69%), 5 cases of Kpn (8.47%), 8 cases of Pae (13.56%), and 3 cases of Pma (5.08%).

**Table 5:** Subgroup analysis for comparison of HARTI patients Disinfection methods (Chlorine disinfectant (500 mg/L)).

Bacteria	All HARTI patients (n = 123)	HARTI patients Disinfection without Chlorine disinfectant (500 mg/L) (n = 104)	HARTI patients Disinfection with Chlorine disinfectant (500 mg/L) (n = 19)
Hin	41 (33.33%)	33 (31.73%)	8 (42.11%)
Sau	3 (2.44%)	3 (2.88%)	0 (0.00%)
Spn	39 (31.71%)	34 (32.69%)	5 (26.32%)
Eco	2 (1.63%)	2 (1.92%)	0 (0.00%)
Aba	3 (2.44%)	1 (0.96%)	2 (10.53%)
Kpn	17 (13.82%)	15 (14.42%)	2 (10.53%)
Pae	14 (11.38%)	12 (11.54%)	2 (10.53%)
Pma	4 (3.25%)	4 (3.85%)	0 (0.00%)

**Table 6:** Subgroup analysis for comparison of HARTI patients Disinfection methods (Alcohol (75%)).

Bacteria	All HARTI patients (n = 123)	HARTI patients Disinfection without Alcohol (75%) (n = 78)	HARTI patients Disinfection with Alcohol (75%) (n = 45)
Hin	41 (33.33%)	26 (33.33%)	15 (33.33%)
Sau	3 (2.44%)	1 (1.28%)	2 (4.44%)
Spn	39 (31.71%)	28 (35.90%)	11 (24.44%)
Eco	2 (1.63%)	1 (1.28%)	1 (2.22%)
Aba	3 (2.44%)	2 (2.56%)	1 (2.22%)
Kpn	17 (13.82%)	7 (8.97%)	10 (22.22%)
Pae	14 (11.38%)	10 (12.82%)	4 (8.89%)
Pma	4 (3.25%)	3 (3.85%)	1 (2.22%)

**Table 7:** Subgroup analysis for comparison of HARTI patients Disinfection methods (Quaternary ammonium salt disinfectant wipes (2500 mg/L)).

Bacteria	All HARTI patients (n = 123)	HARTI patients Disinfection without Alcohol (75%) (n = 64)	HARTI patients Disinfection with Alcohol (75%) (n = 59)
Hin	41 (33.33%)	23 (35.94%)	18 (30.51%)
Sau	3 (2.44%)	2 (3.12%)	1 (1.69%)
Spn	39 (31.71%)	16 (25.00%)	23 (38.98%)
Eco	2 (1.63%)	1 (1.56%)	1 (1.69%)
Aba	3 (2.44%)	3 (4.69%)	0 (0.00%)
Kpn	17 (13.82%)	12 (18.75%)	5 (8.47%)
Pae	14 (11.38%)	6 (9.38%)	8 (13.56%)
Pma	4 (3.25%)	1 (1.56%)	3 (5.08%)

The data in the table clearly indicate the superior disinfection performance of chlorine-containing disinfectants in daily clinical work. At the same time, Hin, Spn, and Kpn are more common in HARTI patients, and precautions should be taken to prevent corresponding infections in ICU clinical work.

## Discussion

ICU patients are frequently susceptible to infections during hospital stays, which can lead to extended hospitalization, complications, and in severe cases, permanent disability or death. Patients in the ICU are particularly vulnerable due to their underlying illnesses, which can compromise their ability to fight off infections, making them more susceptible to acquiring infections [12]. A prospective, multicenter study reported that the probability of hospital-acquired infections in patients staying in the ICU for 7 days or more could rise to over 70% [13]. The most common sites of hospital-acquired infections are the respiratory tract, abdomen, bloodstream, and urinary tract, with respiratory infections accounting for 63.5%. Recent studies suggest that age, BUN, cancer, tracheostomy, and central venous catheters may be potential risk factors for ICU-acquired infections [14–16]. This research thoroughly analyzes potential factors influencing disease prognosis, such as blood markers, medication, and nursing interventions, to assess the reliability of the independent predictive factors mentioned in the article for prognosis evaluation. Additionally, a nomogram is constructed to present these findings, making it more suitable for clinical assessment.

Surface disinfection is one of the effective measures to block nosocomial infections. According to Kumar et al., during the COVID-19 pandemic starting in 2019, the primary pathogen, SARS-CoV-2, could adhere to surfaces in the hospital environment via droplets, posing a potential risk for nosocomial infections [17]. In clinical practice, peracetic acid, hydrogen peroxide, and iodine tincture are also utilized in addition to the three frequently employed surface disinfectants listed in the text [18]. This study focuses on chlorine disinfectant, which recent research has confirmed can effectively reduce bacterial activity. Testing with chlorine dioxide solutions at concentrations of 5–20 mg/L reduced bacterial activity by 98.2%, and antiviral effects against H1N1 and EV71 strains were observed at concentrations of 46.39 mg/L and 84.65 mg/L. Additionally, subtoxic oral tests in drinking water at 40 mg/L showed no significant toxic symptoms [19].

In related research by Li et al., using different concentrations of chlorine-containing disinfectants (500 mg/L, 1000 mg/L, 2000 mg/L) for disinfection of ICU bedside units, it was observed that a 500 mg/L chlorine solution efficiently disinfected and significantly reduced hospital costs [20]. The safety and reliability of chlorine disinfectants are widely recognized. The Centers for Disease Control and Prevention recommend disinfecting surfaces frequently touched by patients with a 5% sodium hypochlorite solution [21]. However, chlorine disinfectants are unstable, and their volatilization can affect the human body through the respiratory tract [22]. Considering these factors, using low-concentration chlorine disinfectant solutions in the ICU can ensure effective surface disinfection while minimizing potential side effects. Chlorine disinfectants, as commonly used surface disinfectants in clinical settings, have been extensively used. Current studies on chlorinated disinfectants are mostly forward-looking studies, and thier safety has been fully confirmed [23–25]. but retrospective study to assess their efficacy have not been seen.

Reducing the occurrence of HARTI in ICU patients is a crucial goal of infection control. Data collected at admission, including the disinfection protocols for surfaces, clinical characteristics, treatment measures, and patient care strategies, can provide multifaceted references for assessing the potential for HARTI in patients. This study has constructed a nomogram based on factors such as Malignant tumor, Admission ICU unit, CRP, APTT, Any norepinephrine use, Blood transfusion, Chlorine disinfectant, and Tracheotomy. The aim is to aid in the early intervention of treatment measures for ICU patients, with the hope that patients will receive effective treatment outcomes and avoid complications during their hospital stay.

This study has several strengths: (1) It is a retrospective study with a substantial amount of data, making the model reliable and easy to apply. (2) The risk factors involved are part of routine information collection, making the results readily obtainable. (3) Both the training and validation models from this study demonstrate good predictive performance. However, the study also has some limitations: (1) It does not track the long-term prognosis of the patients. (2) This is a single-center retrospective study, and there is potential for expanding both the center and the dataset.

## Conclusion

Based on the results of this study, we believe that timely regulation of MONO and BUN values in blood indicators for ICU patients, intervention on corresponding indicators in the SOFA score, and the use of Chlorine disinfectant (500 mg/L) for surface disinfection, with a focus on disinfecting bed railings and micro-infusion pumps, can significantly reduce the incidence of HARTI, allow for early prevention and adjustment of HARI, and simultaneously benefit more patients.
